# Supplementary figures and images for: Genome-Wide Analysis of OPR Family Genes in Cotton Identified a Role for GhOPR9 in Verticillium dahliae Resistance
Source: Genes (Basel). 2020 Sep 27;11(10):1134. doi: 10.3390/genes11101134 (PMC7600627; doi:10.3390/genes11101134)

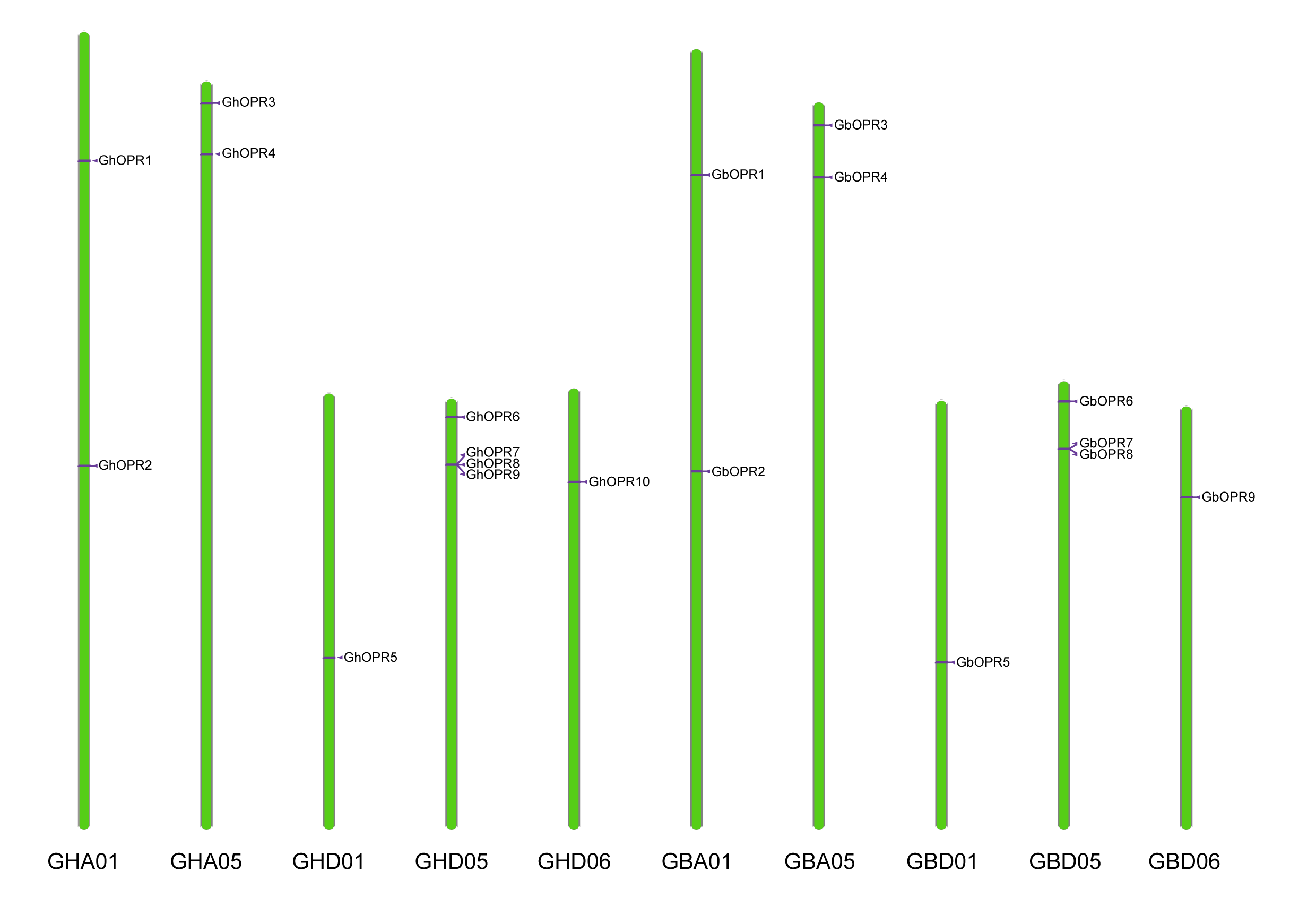

Supplement: Supplementary file 1 [file genes-11-01134-s001.zip › Figure S1 Chromosomal localization of GhOPR and GbOPR genes.tif]

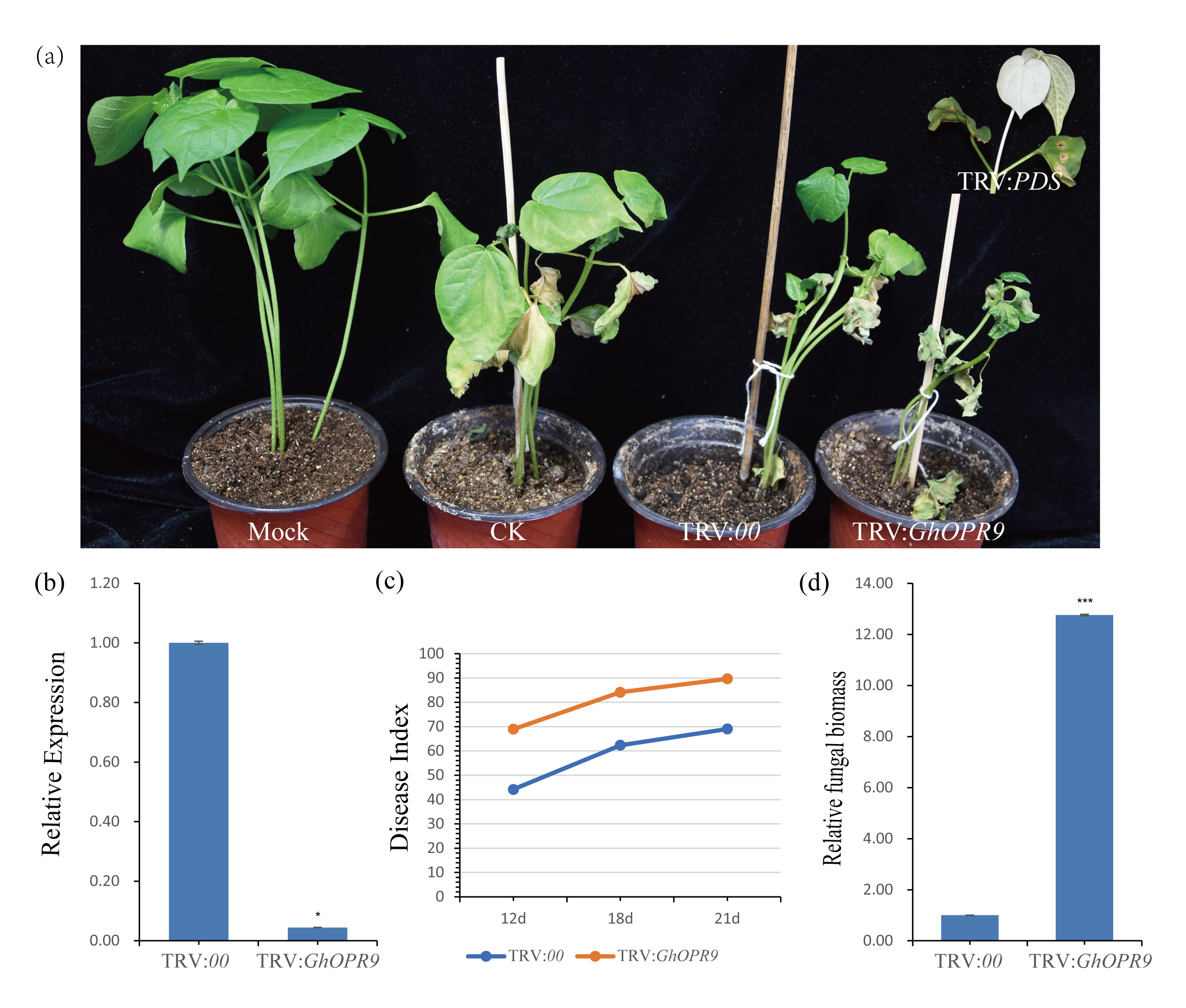

Supplement: Supplementary file 1 [file genes-11-01134-s001.zip › Figure S2 GhOPR9 silencing by virus induced gene silencingpositively regulates cotton resistance against V. dahliae (VIGS) in susceptible G. hirsutum cv. Jimian No.11.tif]

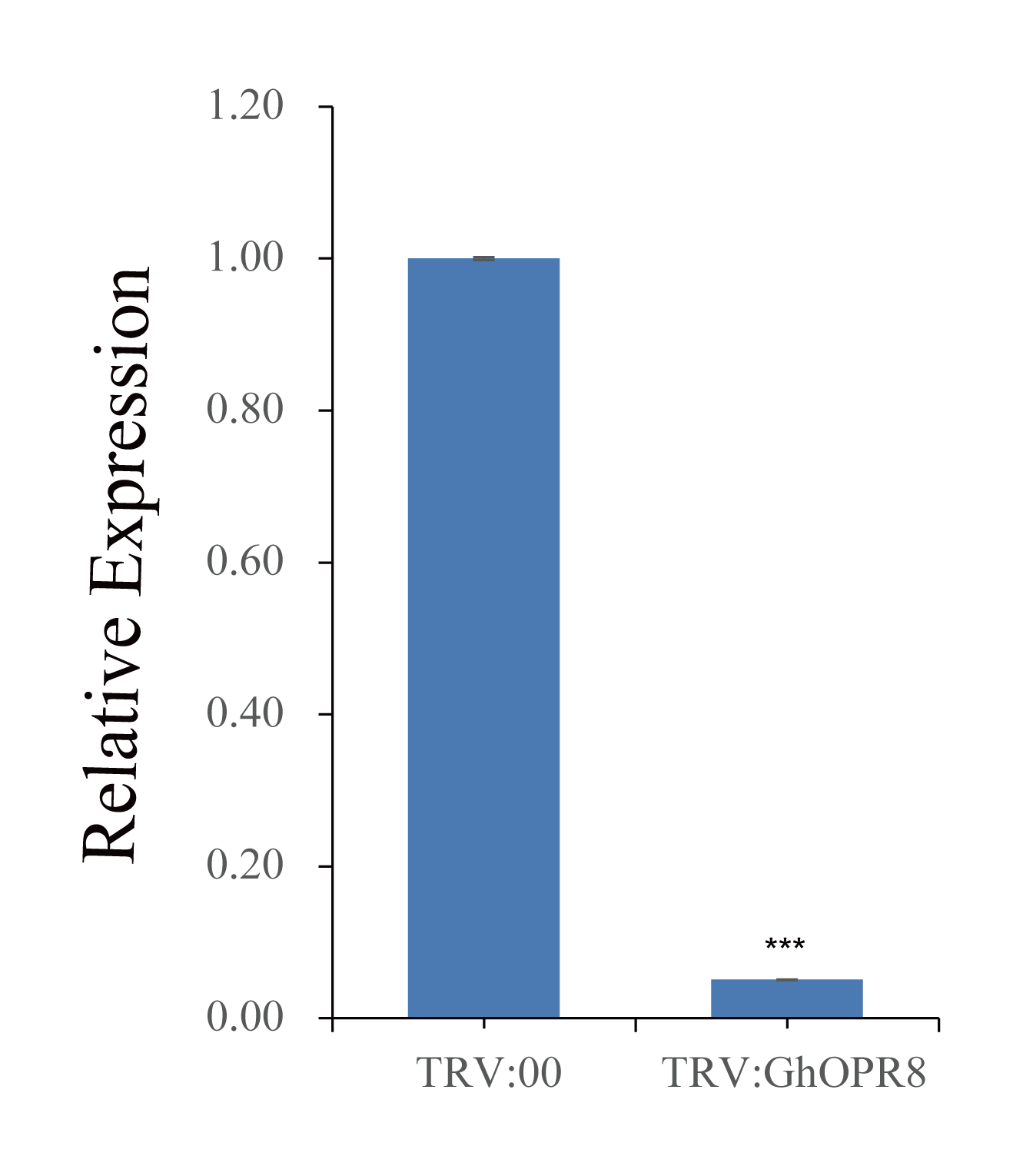

Supplement: Supplementary file 1 [file genes-11-01134-s001.zip › Figure S3 The expression level of GhOPR8 in the TRV00 and TRVGhOPR9 plants Zhongzhimian No.2.tif]
